# Health-related quality of life before planned admission to intensive care: memory over three and six months

**DOI:** 10.1186/1477-7525-8-103

**Published:** 2010-09-16

**Authors:** Maurizia Capuzzo, Sara Bertacchini, Elena Davanzo, Giovanna Felisatti, Laura Paparella, Laura Tadini, Raffaele Alvisi

**Affiliations:** 1 University Section of Anaesthesiology and Intensive Care, Azienda Ospedaliero-Universitaria di Ferrara Arcispedale S. Anna, Ferrara, Italy; 2 Department of Medical and Surgical Specialties, University Hospital of Florence, Florence, Italy; 3 Department of Medicine, Surgery, and Critical Care, Section of Anaesthesiology and Intensive Care, University Hospital of Florence, Florence, Italy

## Abstract

**Background:**

The validity of Health-Related Quality of Life (HRQOL) recalled by ICU admitted patients have not been published. The aim of this study was to compare the baseline HRQOL measured before surgery and ICU admission with that recalled at 3 and 6 months in a population of patients with planned ICU admission after surgery.

**Methods:**

This prospective study was performed in three Italian centres on patients who had undergone General, Orthopaedic or Urologic surgery. All adult patients with planned ICU admission between October 2007 and July 2008 were considered for enrolment. At hospital admission, the Mini Mental Status Examination and EuroQoL (EQ) questionnaire (referring to the last two weeks) were administered to the patients who consented. Three and six months after ICU admission, the researchers administered by phone the EQ questionnaire and Post-Traumatic Stress Syndrome 14 questions Inventory, asking the patients to rate their HRQOL before surgery and ICU admission. Past medical history demographic and clinical ICU-related variables were collected.

**Statistical analysis:**

Chi-square test and non parametric statistics were used to compare groups of patients. The EQ-5D was transformed in the time trade-off (TTO) to obtain a continuous variable, subsequently analysed using the Intraclass Correlation Coefficient (ICC).

**Results:**

Of the 104 patients assessed at baseline and discharged from the hospital, 93 had the EQ administered at 3 months, and 89 at 6 months. The ICC for TTO recalled at 3 months vs pre-ICU TTO was 0.851, and that for TTO recalled at 6 months vs pre-ICU TTO was 0.833. The ICC for the EQ-VAS recalled at 3 months vs pre-ICU EQ-VAS was 0.648, and that for the EQ-VAS recalled at 6 months vs pre-ICU EQ-VAS was 0.580. Forty-two (45%) patients assessed at 3 months gave the same score in all EQ-5D items as at baseline. They underwent mainly orthopaedic surgery (p 0.011), and perceived the severity of their illness as lower (p 0.009) than patients scoring differently at 3 months in comparison with baseline.

**Conclusions:**

The patients with planned ICU admission have a good memory of their health status as measured by EQ-5D in the period preceding surgery and ICU admission, especially at three months.

## Background

Health-related Quality of Life (HRQOL) of the patients admitted to Intensive Care Unit (ICU) is one of the most relevant outcome measures for patients, families, physicians and society. To understand the clinical meaning of HRQOL in ICU survivors, we should make comparisons, either with the HRQOL of the matched general population or with the patient HRQOL before ICU admission [1].

Considering that baseline HRQOL of ICU patients has been shown to be significantly lower than that of the matched general population [2-5], it appeared wise for researchers to compare post-ICU with baseline HRQOL [2-4,6]. However, most of the ICU admissions are unpredictable, so baseline HRQOL can be measured only *a posteriori *in those patients who are asked about their HRQOL in the period of two [7] or three [6,8] months before ICU admission. Nevertheless, asking patients to recall and rate a previous HRQOL may introduce a recall bias since patients may not accurately remember their status prior to critical illness [9-11], their evaluation being influenced by the present status. We have only found one study considering patients admitted to the hospital with chest pain where researchers assessed the ability of respondents to recall their pre-hospital admission HRQOL [12]. In that study, six generic health status questionnaires were self-administered to the patients during hospital stay and mailed home three months after hospital discharge. The assessments were generally similar, but some patients reported that they were more functional before ICU admission in mental well-being, work and housework performance at the assessment performed at three months than in that performed during hospital stay [12]. Nothing is known about the "memory stability" of baseline HRQOL in patients admitted to ICU.

Some patients undergoing scheduled surgical procedures are admitted to ICU due to their poor clinical conditions and/or to type and magnitude of surgery. They are a group of ICU patients suitable for the on time assessment of HRQOL before ICU admission.

Therefore we designed a study to compare the baseline HRQOL measured before surgery and ICU admission with that recalled at 3 and 6 months in a population of patients with planned ICU admission after general, orthopaedic and urologic scheduled surgery.

## Methods

The study was performed in three Italian hospitals on patients who had undergone General and Orthopaedic and Urologic surgeries, respectively. The Hospital Ethics Committees approved the study protocol and written consent was obtained from the enrolled patients. All consecutive adult patients where ICU admission was planned at the time of the anaesthetic visit between October 2007 and July 2008 were considered for enrolment. The criteria for inclusion in the study were age > 18 years, ability to co-operate and consent to the study. Patients not aware of self and environment were excluded, as well as those refusing to participate.

At the preoperative anaesthetic visit of a patient who was a potential candidate for ICU postoperative admission, the physician informed the patient about the study. At hospital admission, the researchers administered the Mini Mental Status Examination [13] and EuroQoL questionnaire [14,15] referring to the last two weeks to the patients who consented (pre-ICU assessment). Additionally, a structured form was used to collect information about the following variables: gender, age, number of years of education, smoking habits (never smoker, former smoker and current smoker), alcohol habits (not used, only occasionally, daily), regular taking of benzodiazepines, beta-blockers, and antihypertensive drugs. Moreover, the following information was collected for each patient: type of surgery and anaesthesia, a severity of illness score (Simplified Acute Physiology Score SAPS II [16]), length of stay (LOS) as number of days in ICU and in hospital after ICU discharge, number of hours on mechanical ventilation, analgesic and sedative drugs administrated during ICU stay, presence of delirium, assessed by the Confusion Assessment Method for the Intensive Care Unit [17] and number of days in delirium.

Three and six months after ICU admission, the same researcher who administered the EuroQoL questionnaire in hospital administered it by phone, asking the patients to rate their HRQOL before surgery and ICU admission. Then, the patients were asked whether their present health status was the same, better, or worse compared with that before surgery and ICU admission.

Moreover, during the same phone call, the researchers administered the Post-Traumatic Stress Syndrome 14 questions Inventory [18].

A minimum of 22 patients per centre were required assuming correlation coefficients would be obtained of over 0.75 with a significance level of 0.01 and a power of 0.80. Considering a projected 10% loss or withdrawal rate, each centre was invited to collect at least 30 patients.

## Instruments used in the study

### Mini Mental Status Examination

The Mini Mental Status Examination (MMSE) was administered to evaluate global cognitive functions, such as orientation in space and time, concentration and attention span, immediate and delayed verbal memory, constructive praxis and language [13]. The final score was adjusted according to the classes of age and education [19]. The results of the MMSE are expressed as a score ranging from 0 to 30.

### EuroQol

The questionnaire administered was EuroQol (EQ). It is a generic questionnaire, easy to administer and consists of two parts. In the first part (EQ-5D), five dimensions (mobility, self-care, usual activities, pain/discomfort, and anxiety/depression) are considered, and, for each, a question is posed with three possible answers: no problems; some/moderate problems; severe/extreme problems. A health state is a combination of one level for each dimension, with 243 possible health states. Preferences have been assessed using time trade-off (TTO) of a subset of health states from a UK population [20]. In the reworked TTO scale the logically best health state (no problem in any of the five dimensions) has the value of 1, while death has the value 0 [21]. Nevertheless, due to the possible presence of a negative factor in the model, there are also states with values lower than 0. In the second part of the EQ (EQ-VAS), the patients are asked to rate their health status on a scale from 100 (the best imaginable health status) to 0 (the worst imaginable health status).

The validity and reliability of the EQ have been tested in the ICU population, and it has been recommended for use in critical care [11]. It was designed for self-completion [15] but it was also administered by telephone [6,22] or by direct interview [23].

Because the EQ VAS was administered by telephone, the results could not be graphically represented on a 20-cm line, as originally proposed. Therefore, EQ VAS was recorded as a numerical rating from 100 (best health status) to 0 (worst health status).

### Confusion Assessment Method for the Intensive Care Unit

The Confusion Assessment Method for the Intensive Care Unit (CAM-ICU) [17] assesses the presence or the absence of the following four features: 1) acute onset of mental status changes or a fluctuating course; 2) inattention; 3) disorganized thinking; 4) altered level of consciousness (i.e. other than alert). The patients are diagnosed as having delirium (i.e. CAM positive) if both features 1 and 2 and either feature 3 or 4 are present.

The CAM-ICU can be administered by doctors or nurses. It has been developed to be used in mechanically ventilated patients and is one of the most commonly used instruments for delirium [24].

### Post-Traumatic Stress Syndrome 14 questions Inventory

The Post-Traumatic Stress Syndrome 14 questions Inventory (PTSS-14) [18] is composed of two parts: part A (assessment of traumatic memories from the ICU) and part B (post-traumatic stress disorder symptoms). Part A of the questionnaire consists of a structured survey asking for possible traumatic experiences during ICU treatment (patient's subjective memory of respiratory distress/dyspnoea, feelings of severe anxiety/panic, severe pain, or nightmares). Patients are asked to answer whether (yes or no) they remember each of four items. Part B evaluates 14 PTSD symptoms (sleep problems, nightmares, depression, jumpiness, need for withdrawal, irritability, frequent mood swings, bad conscience, fear of place and situation, muscular tension, upsetting/unwanted thoughts or image of the time on ICU, feeling numb, avoiding places/people or situations that remind them of the ICU, feeling as though plans or dreams for the future will not come true). When completing the questionnaire, the patients rate their symptoms using a scale from 1 (never) to 7 (always) and sum score ranging from 14 to 98 points is calculated. A total score of more of 45 points has been reported to be predictive of PTSD [18].

### Perceived severity of illness

The severity of illness as perceived by the patient was assessed at 3 months using a verbal Numerical Rating Scale (NRS) ranging from 0 to 10. The investigator asked the patient to indicate the perceived level of his/her severity of illness at the time of ICU stay, saying "Please, tell me how serious your clinical conditions were while in ICU, using a scale where 0 means «not serious» and 10 means «as serious as possible».

### Statistical Analysis

The data are expressed as median with Inter Quartile Range (IQR). Categorical variables are described as absolute numbers with percentages. Statistical analysis was carried out using a software package (SPSS 11.5 Chicago, Illinois, USA) and two-tailed p-values less than 0.05 were selected as significant. The Chi-square test, or Fisher Exact test, when appropriate, was used for categorical variables. The Kolmogorov-Smirnov test showed that most of the continuous variables were not normally distributed, so they were analysed using Mann-Whitey and Kruskall-Wallis statistics.

To evaluate the reliability of the patients' memory of HRQOL at 3 and 6 months after ICU admission, in comparison with that assessed before surgery and ICU admission, we transformed the EQ-5D in the time trade-off (TTO) as assessed according to a subset of health states from a UK population [20]. This made the EQ-5D a continuous variable, which was subsequently analysed using the Intraclass Correlation Coefficient (ICC), two-way mixed average measures model (consistency). The EQ-VAS, which was also a continuous variable, was analysed in the same way. The ICC measures agreement from 0 or less (no agreement) to 1 (perfect agreement), with a good to excellent agreement for values > 0.6 according to the Fleiss' rules [25].

A forward stepwise logistic regression analysis was performed to determine which variables pertinent to the patients were independently associated with the same rating of HRQOL before surgery and ICU admission, and at 3 months. To make the dependent variable categorical the comparison between the EQ-5D scored before surgery and ICU admission and that scored at 3 months was categorized as the "same" when there was no difference between any items, and "different" when at least one EQ-5D item at 3 months was different in comparison with that before surgery and ICU admission. Factors that were significant for a p value < 0.20 in the univariate analyses were entered into the multivariate stepwise logistic regression analysis. Odds ratio were estimated from b coefficients and expressed with 95% Confidence Interval (95% CI).

## Results

**Figure 1:**
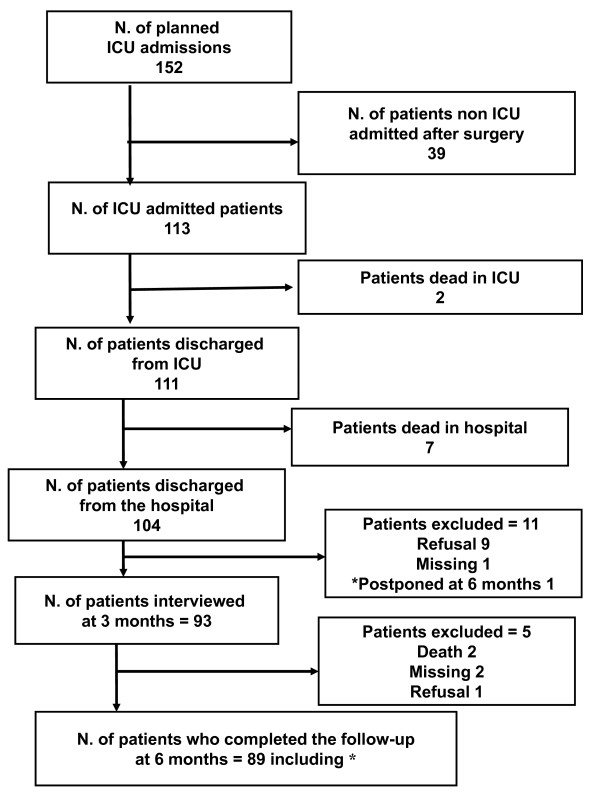
**Flowchart of studied patients**.

During the study period 152 patients undergoing surgery and planned to be admitted to the study ICUs consented to participate. Of these, 39 were not subsequently admitted to the ICU due to a less aggressive surgical procedure than previously supposed. Of the remaining 113 patients, 2 died in ICU and 7 died in hospital after discharge. Therefore 104 patients admitted to ICU after planned surgery were discharged from the hospital. Nine of them refused to take part in the subsequent phase of the study, one was lost despite multiple attempts to find her and one was admitted to another hospital and was only administered the questionnaire after 6 months. The final group consisted of 93 patients having the EQ questionnaire administered at 3 months and 89 at 6 months (flow-chart in figure 1).

**Figure 2:**
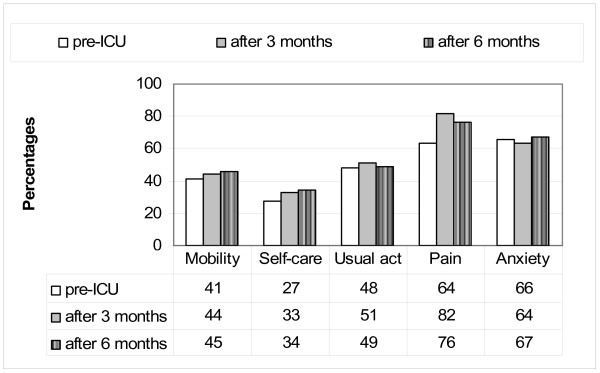
**Percentages of patients reporting any problems in EuroQol-5D at the pre-ICU assessment, and recalling any problems at the assessments performed at 3 and 6 months**.

The 94 patients included at any time in the study underwent the following kind of surgery: general including major gastrointestinal surgery (14 patients), thoracic surgery (5), esophagectomy (4), and abdominal aortic surgery (2); orthopaedic including hip prosthesis (20 patients), knee prosthesis (6) and major osteosynthesis (7); and urologic including nephrectomy (13 patients), cystectomy (9), prostatectomy (9) and other (5). The demographic and clinical characteristics of the study patients are reported in table 1. The mean TTO according to the EQ-5D assessed at the time of the preoperative visit was 0.596 (95% CI 0. 535-0.658), the mean TTO recalled at 3 months was 0.581 (95% CI 0.522-0.639), and that recalled at 6 months was 0.601 (95%CI 0.544-0.658). The ICC for TTO recalled at 3 months vs pre-ICU TTO was 0.851, and that for TTO recalled at 6 months vs pre-ICU TTO was 0.833. The mean EQ-VAS assessed at the time of the preoperative visit was 48.7 (95% CI 45.7-51.7), that recalled at 3 months was 49.4 (95% CI 45.9-52.8), and that recalled at 6 months was 51.6 (95%CI 47.8-55.3). The ICC for the EQ-VAS recalled at 3 months vs pre-ICU EQ-VAS was 0.648, and that for the EQ-VAS recalled at 6 months vs pre-ICU EQ-VAS was 0.580. The percentages of patients reporting any problems in EuroQol-5D at the pre-ICU assessment, and recalling any problems at the assessments performed at 3 and 6 months are reported in Figure 2.

**Table 1:** Demographic and clinical characteristics of the study patients

Number of patients	93	
Number of males	64	68.8%
Age (years) median (IQR)	74	(66-78)
Education (years): < 8	33	35.4%
8-13	53	60.6%
> 13	7	7.5%
MMSE adjusted median (IQR)	26	(25-28)
Use of any benzodiazepines	11	11.8%
Use of any antidepressants	8	8.6%
Use of antihypertensive drugs	78	83.8%
Use of B-blockers	22	23.6%
Use of any statins	24	25.8%
Smoking habits: no	25	26.9%
former	55	59.1%
yes	13	14.0%
Alcohol: no	49	52.6%
sometimes	31	33.4%
every day	13	14.0%
Type of surgery: General	24	25.8%
Orthopaedic	33	35.4%
Urologic	36	38.8%
Type of anaesthesia: regional ± general	19	20.5%
general	74	79.5%
**ICU and hospital course**		
SAPS II median (IQR)	29	(24-43)
Number of patients ventilated	41	44.0%
Duration of ventilation (h) median (IQR)	6	(4-19)
Number of patients with delirium in ICU	6	6.4%
ICU LOS^a ^(days) median (IQR)	2	(1-3)
Hospital LOS^a ^(days) median (IQR)	7	(5-10)

^a ^LOS: Length of stay

To investigate the effect of prolonged ICU LOS on recall, the reliability of EQ-5D and EQ VAS recalled at 3 months by the 64 patients with an ICU LOS lower or equal to the median value (2 days) and by the 29 patients staying in ICU more than 2 days were analysed. The demographic and clinical characteristics of those two group patients are reported in table 2. In the patients with ICU LOS ≤ 2 days, the ICC for TTO recalled at 3 months vs pre-ICU TTO was 0.872, and that for TTO recalled at 6 months vs pre-ICU TTO was 0.832. The median EQ-VAS assessed at the time of the preoperative visit was 50 (IQR 40-50), that recalled at 3 months was 50 (IQR 40-50), and that recalled at 6 months was 50 (IQR 40-60). The ICC for the EQ-VAS recalled at 3 months vs pre-ICU EQ-VAS was 0.612, and that for the EQ-VAS recalled at 6 months vs pre-ICU EQ-VAS was 0.569. In the patients with ICU LOS > 2 days, the ICC for TTO recalled at 3 months vs pre-ICU TTO was 0.765, and that for TTO recalled at 6 months vs pre-ICU TTO was 0.823. The median EQ-VAS assessed at the time of the preoperative visit was 50 (IQR 40-60), that recalled at 3 months and that recalled at 6 months were exactly the same. The ICC for the EQ-VAS recalled at 3 months vs pre-ICU EQ-VAS was 0.698, and that for the EQ-VAS recalled at 6 months vs pre-ICU EQ-VAS was 0.765.

**Table 2:** Characteristics of the patients with ICU length of stay (LOS) ≤ 2 and > 2 days

ICU length of stay	≤ 2 days	> 2 days	p
Number of patients (%)	64 (69)	29 (31)	
Number of males (%)	41 (64)	23 (79)	0.157
Age, y: median (IQR)^a^	74 (66-79)	74 (66-77)	0.584
Education (%):			
< 8 years	17 (27)	16 (55)	
≥8 years	47 (73)	13 (45)	
Use of any benzodiazepines (%)	8 (12)	3 (10)	1
Use of any antidepressants (%)	5 (8)	3 (10)	0.701
Use of any antihypertensive drugs (%)	55 (86)	23 (79)	0.544
Use of any B-blockers (%)	15 (23)	7 (24)	1
Use of any statins (%)	14 (22)	10 (34)	0.211
Smoking habits (%): no	36 (56)	19 (65)	0.453
Alcohol (%): no	22 (34)	9 (31)	0.656
Type of surgery (%):			
General	9 (14)	10 (34)	0.064
Orthopaedic	28 (44)	8 (27)	
Urologic	27 (42)	11 (38)	
Type of anaesthesia (%): regional ± general	13 (21)	6 (21)	0.814
general		51 (79)	23 (79)
**ICU and hospital course**			
SAPS II median (IQR)^a^	29 (23-39)	31 (26-44)	0.309
Mechanical Ventilation in ICU (%)	24 (37)	17 (58)	0.073
Mechanical Ventilation (hours) median (IQR)^a^	8 (3-8)	42 (13-88)	0.009
Delirium in ICU (%)	0	6 (20)	0.001
ICU LOS^a ^(days) median (IQR)^a^	1 (1-2)	1 (1-3)	< 0.001
Hospital LOS (days) median (IQR)^a^	6 (5-8)	9 (6-14)	0.004
**Follow-up at 3 months**			
Perceived severity of illness > 5 (%)^b^	14 (22)	9 (31)	0.437
PTSS-14 median (IQR)^a^	23 (18-30)	26 (20-33)	0.088
HRQOL Comparison: worse (%)	21 (33)	13 (45)	0.378
**Follow-up at 6 months (89 patients)**			
Perceived severity of illness > 5 (%)^b^	9 (14)	8 (27)	0.072
PTSS-14 median (IQR)^a^	23 (18-30)	26 (20-33)	0.001
HRQOL Comparison: worse (%)	16 (25)	9 (31)	0.438

P: statistical significance according to chi square test, except for age, SAPS II, ICU and Hospital length of stay, and PTSS-14 (Mann Withney test).

^a ^IQR: Inter Quartile Range

^b ^Perceived severity of illness assessed by Numerical Rating Scale 0 to 10.

**Figure 3:**
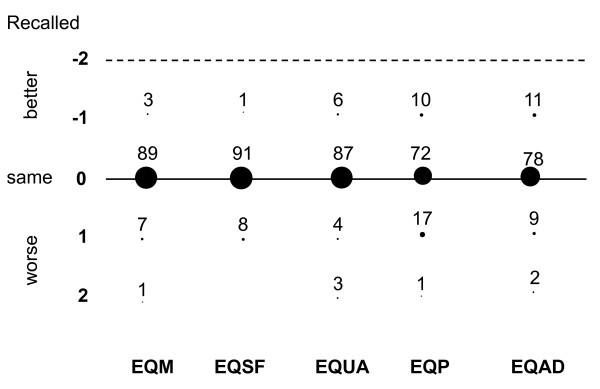
**Differences between pre-ICU and 3-month recalled EuroQol-5D**. Numbers are percentages of patients. EQ: EuroQol-5D. EQM mobility, EQSF self-care, EQUA usual activities, EQP pain/discomfort, EQAD anxiety/depression

**Figure 4:**
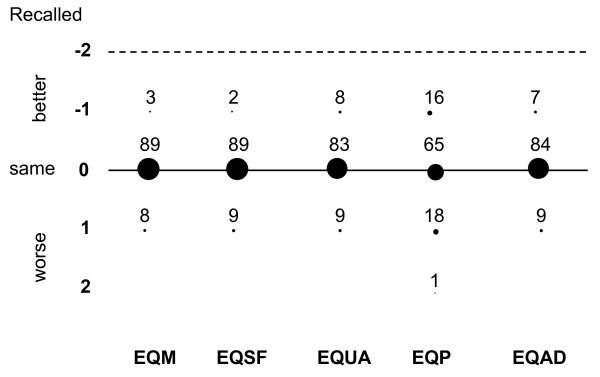
**Differences between pre-ICU and 6-month recalled EuroQol-5D**. Numbers are percentages of patients. EQ: EuroQol-5D. EQM mobility, EQSF self-care, EQUA usual activities, EQP pain/discomfort, EQAD anxiety/depression

The percentages of patients who gave the same answer at 3 months as that given pre-ICU were 89% for EQ-5D dimension of mobility, 91% for self-care, 87% for usual activities, 72% for pain/discomfort, and 78% for anxiety/depression. Similar results were found for the answers given at 6 months, with percentages of 89% for mobility and for self care, 83% for usual activities, 65% for pain/discomfort and 84% for anxiety/depression, respectively. The differences between pre-ICU EQ-5D recorded at 3 months and EQ-5D given before ICU admission and between pre-ICU EQ-5D recorded at 6 months and EQ-5D given before ICU admission, for each dimension are reported in Figure 3 and 4, respectively. In the figures, the differences were calculated for each item as the value remembered at 3 months minus the value given before ICU admission: for instance, a patient who remembered having an EQ-5D for mobility of 1 (no problems) at 3 months and scored 2 (some problems) before ICU admission was considered as having a difference of -1, meaning that he/she recalled a better past mobility than that previously assessed.

Out of the 93 patients assessed at 3 months, 42 (45%) gave the same score in all EQ-5D items as before surgery and ICU admission. At the univariate analysis (Table 3), more patients who reported at 3 months the same scoring in all EQ-5D items as before surgery and ICU admission underwent orthopaedic surgery (p 0.011) and perceived the severity of their illness lower or equal 5 (p 0.009) than the patients scoring differently at 3 months in at least one EQ-5D item.

**Table 3:** Characteristics of the patients assessed at 3 months according to the comparison with the score given before surgery and ICU admission

EQ-5D at 3 months vs pre-admission	same	different	p
Number of patients (%)	42 (45)	51 (55)	
Number of males (%)	28 (67)	36 (71)	0.822
Age, y: median (IQR)^a^	73 (61-80)	74 (67- 78)	0.389
Education (%):			
Education (%): < 8 years	12 (29)	21 (41)	0.295
≥8 years	30 (72)	30 (59)	
Use of any benzodiazepines (%)	3 (7)	8 (16)	0.334
Use of any antidepressants (%)	6 (14)	2 (4)	0.134
Use of any antihypertensive drugs (%)	36 (86)	42 (82)	0.780
Use of any B-blockers (%)	6 (14)	16 (31)	0.085
Use of any statins (%)	11 (26)	13 (25)	1
Smoking habits (%): no	12 (29)	13 (25)	0.921
Alcohol (%): no	25 (60)	24 (47)	0.322
Type of surgery (%):			
General	5 (12)	19 (37)	0.011
Orthopaedic	20 (48)	13 (26)	
Urologic	17 (40)	19 (37)	
Type of anaesthesia (%): regional ± general	11 (26)	8 (16)	0.302
general	31 (74)	43 (84)	
**ICU and hospital course**			
SAPS II median (IQR)^a^	30 (24-45)	29 (24-39)	0.591
Mechanical Ventilation in ICU (%)	15 (36)	26 (51)	0.150
Delirium in ICU (%)	1 (2)	5 (10)	0.214
ICU LOS (days) median (IQR)^a^	2 (1-2)	2 (1-3)	0.203
Hospital LOS^a ^(days) median (IQR)^a^	7 (5-9)	7 (5-10)	0.590
**Follow-up at 3 months**			
Perceived severity of illness > 5 (%)^b^	5 (12)	18 (35)	0.009
PTSS-14 median (IQR)^a^	25 (18-31)	24 (20-31)	0.685
HRQOL Comparison: worse (%)	14 (33)	20 (39)	0.711

In the comparison between EQ-5D pre-admission and at 3 months "same" means that the scores at 3 months were the same while "different" means that at least one score at 3 months was different in at least one EQ-5D item in comparison with that before surgery and ICU admission.

P: statistical significance according to chi square test, except for age, SAPS II, ICU and Hospital length of stay, and PTSS-14 (Mann Withney test).

^a ^IQR: Inter Quartile Range

^b ^Perceived severity of illness assessed by Numerical Rating Scale 0 to 10.

Two of the variables entered in the logistic regression analysis (use of antidepressants, use of beta-blockers, General, Orthopaedic or Urologic surgery, mechanical ventilation while in ICU, and perceived severity of illness categorized as ≤ or > 5 in NRS 0-10) were significantly associated with reliability of EQ-5D assessment at 3 months (lack of any difference in all items between the EQ-5D assessed before surgery and ICU admission and that recalled 3 month after ICU discharge). The general surgery patients showed a significantly poorer ability to recall pre-ICU EQ-5D (Odds Ratio 0.192 with 95%CI 0.062-0.590; p 0.004). The chronic use of beta-blockers was directly associated with better ability to recall pre-ICU EQ-5D (Odds Ratio 3.457 with 95%CI 1.159-10.313; p 0.026). To investigate whether the ICU LOS (≤ or > 2 days) influences the pre-ICU EQ-5D recall, we tried to include this variable in the model, but it was not relevant.

The patients were grouped also according to the answers given at 6 months in comparison with those given before ICU admission: 41 gave same answers and 48 gave different answers. The univariate analysis showed that the following variables were different at a p level < 0.20: gender female (p 0.112); use of benzodiazepine (p 0.170); use of beta-blockers (0.014); no alcohol habit (0.152); type of surgery (p 0.007); SAPS II with a cut-off value of 30 (p 0.057) and perceived severity of illness at 6 months with 5 as cut-off (p0.023). A new logistic regression analysis was performed using those variables and three variables were included in the final model. Both the general surgery and urologic surgery patients showed a significantly poorer ability to recall pre-ICU EQ-5D (Odds Ratio 0.144 with 95%CI 0.039-0.525; p 0.003, and Odds Ratio 0.328 with 95%CI 0.116-0.922; p 0.035, respectively). The chronic use of beta-blockers was again directly associated with better ability to recall pre-ICU EQ-5D (Odds Ratio 4.431 with 95%CI 1.373-14.304; p 0.013).

## Discussion

This is the first study demonstrating that the patients with planned ICU admission assessed after three months generally have a good memory of their health status as measured by EQ-5D in the period preceding surgery and ICU admission. This memory also appears to remain good after 6 months, because the values of ICC for EQ-5D recalled at 3 months and for EQ-5D recalled at 6 months in comparison with the pre-ICU EQ-5D were both higher than 0.8, which is generally regarded as an excellent concordance [25]. On the other hand, the ICC for the EQ-VAS recalled was just acceptable at 3 months (0.648) and became lower at 6 months in comparison with the pre-ICU level (0.580). The reason for the different behaviour of EQ-5D and EQ-VAS may be strictly mathematical because the former is based on three possible answers (no problems; some/moderate problems; severe/extreme problems) for each of the EQ-5D items, while the latter is on a 101 point scale: the larger the scale parameter, the more spread out the distribution, and the higher the probability of making a different choice.

As far as methodological aspects are concerned, the study hospitals were located in two contiguous and similar Regions of Northern Italy, and the instrument used has been adopted in studies investigating different populations [26-28]. The TTO transformation of patients' EQ-5D was performed using data from a UK population, so those for Italian people may be different. However, considering that the transformation was just used to analyse the statistical agreement - the concordance between the ratings of the same thing and period assessed at different points in time - also different formulas to obtain TTO applied to all EQ-5D ratings would have given the same concordance.

Our findings agree with those of Guadagnoli et al. [12] who studied 1038 chest pain patients admitted to six hospitals for actual or suspected acute myocardial infarction and found substantial stability over time in response to individual items. The average difference between the scores assessed at the two times was significantly different from zero in only two cases; in both cases, patients reported that they were more functional before admission when asked at 3 months than when asked at the time of hospital stay. Accordingly, the EQ-VAS of our patients showed a slight trend towards increasing over time, suggesting that previous health status may be perceived better as time passes.

The information given by our study may be more useful than expected. In fact, most ICU admissions are unpredictable, so baseline HRQOL is usually measured according to the relatives' opinions [29]. Nevertheless, proxies may not accurately provide baseline measurements due to stress, infrequent contact with the patient, or different perceptions in comparison with the patient [30,31]. Diaz-Prieto et al [32] found kappas for patient-proxy concordance ranging from 0.52 for mobility to 0.31 for anxiety/depression, without the effect of the type of patient/proxy relationship, or level of education or admission category (trauma, scheduled or unscheduled surgery, or medical). On the other hand, in the same study EQ-5D VAS scores obtained from patients and proxies correlated much better, with an ICC coefficient of 0.72, which is not so far from that found in the present study (0.648 at three months). Therefore, investigators interested in the before/after comparison of the quality of life of ICU patients may obtain a more reliable assessment of baseline health status interviewing the patients three or six months after discharge than interviewing the relatives.

The multivariate analysis showed that the ability to recall pre-ICU EQ-5D was poorer for general surgery patients at 3 and 6 months, and for urologic surgery patients at 6 months. Possibly, the sequelae of surgery or anti-neoplastic treatments, if required, may affect HRQOL memory in those patients, in comparison with orthopaedic surgery patients.

The similar ability to recall pre-ICU EQ-5D and EQ VAS showed by the patients with ICU LOS ≤ and > 2 days suggests a limited effect of ICU stay on recall and gives strength to our study, despite the significant differences between the two patient groups in the incidence of delirium, hospital LOS and PTSS-14 at 6 months (Table 2). Considering 45 as cut-off for PTSS-14 [18], only patients with ICU LOS > 2 days had high values (one with 45 at 3 months and 24 at 6 months, and two with 45 and 50, respectively, at 6 months). Accordingly, we cannot exclude that the development of any PTSD symptoms may affect the recall of pre-ICU HRQOL. Interestingly, the chronic use of beta-blockers was associated with better ability to recall pre-ICU EQ-5D, both at 3 and at 6 months. This findings agrees with a recent study showing that a pharmacological blockade of beta-adrenoceptors prevents glucocorticoid-induced memory retrieval deficits in human subjects [33]. A number of studies have examined the influence of giving a β-adrenergic receptor antagonist [34,35], to try to reduce the incidence of PTSD, however these therapies may be problematic in the critical care population and more research is needed to clarify their role.

As far as study limitations are concerned, our aim was to investigate stability of memory of HRQOL. Therefore, the only population suitable for the on time assessment before ICU admission consisted of patients with planned ICU admission. Consequently, it does not demonstrate that the findings reported are of value for patients with unplanned ICU admissions. Considering that Diaz-Prieto et al [32] found no relationship between patient-proxy concordance and admission category, we may infer that our findings should be of general value. The exclusion of patients who were not admitted to ICU after surgery despite an admission planned at the time of the anaesthetic visit, allowed a homogeneous sample of patients with the same factors possibly influencing patient memory to be evaluated. In fact, the administration of analgesic and sedatives, which is a common ICU practice, has been demonstrated to influence patient memory of the ICU stay [36,37]. This practice may also influence the memory of the period preceding ICU admission, so we preferred to study the patients exposed to the same risk factors, that is those really admitted to ICU.

## Conclusions

Patients with planned ICU admission have a good memory of their health status in the period preceding surgery and ICU admission. Their recall of EQ-5D appears to be good both at three and six months, being similar in the patients with different length of stay in ICU (≤ or > 2 days). Investigators may rely on the ICU patients' memory at 3 months.

## Competing interests

The authors declare that they have no competing interests.

Some data have been reported in the thesis of Specialization in Anaesthesia and Intensive Care of one of the authors (GF) and the thesis won the award "Concorso Avant-Garde 2009" of the University of Pisa.

## Authors' contributions

MC and SB conceived and designed the study. ED, GF, LP, and LT managed organisation and data collection. Data analysis was performed by MC, SB, ED, and GF. MC, SB, ED and GF wrote the draft of the report. All the authors contributed to the final writing of the report. RA performed the critical revision of the manuscript and supervision.

All authors read and approved the final manuscript.
